# Assessing the Selectivity of FXR, LXRs, CAR, and RORγ Pharmaceutical Ligands With Reporter Cell Lines

**DOI:** 10.3389/fphar.2020.01122

**Published:** 2020-07-24

**Authors:** Lucia Toporova, Marina Grimaldi, Abdelhay Boulahtouf, Patrick Balaguer

**Affiliations:** Institut de Recherche en Cancérologie de Montpellier (IRCM), INSERM U1194, ICM, Univ Montpellier, Montpellier, France

**Keywords:** reporter cell lines, nuclear receptors, basal activity, selectivity, agonism, antagonism

## Abstract

To characterize human nuclear receptor (NR) specificity of synthetic pharmaceutical chemicals we established stable cell lines expressing the ligand binding domains (LBDs) of human FXR, LXRα, LXRβ, CAR, and RORγ fused to the yeast GAL4 DNA binding domain (DBD). As we have already done for human PXR, a two-step transfection procedure was used. HeLa cells stably expressing a Gal4 responsive gene (HG5LN cell line) were transfected by Gal4-NRs expressing plasmids. At first, using these cell lines as well as the HG5LN PXR cells, we demonstrated that the basal activities varied from weak (FXR and LXRs), intermediate (PXR), to strong (CAR and RORγ), reflecting the recruitment of HeLa co-regulators in absence of ligand. Secondly, we finely characterized the activities of commercially available FXR, LXRα, LXRβ, CAR, RORγ, and PXR agonists/antagonists GW4064, feraxamine, DY268, T0901317, GW3965, WAY252623, SR9238, SR9243, GSK2033, CITCO, CINPA1, PK11195, S07662, SR1078, SR0987, SR1001, SR2211, XY018, clotrimazole, dabrafenib, SR12813, and SPA70, respectively. Among these compounds we revealed both, receptor specific agonists/antagonists, as well as less selective ligands, activating or inhibiting several nuclear receptors. FXR ligands manifested high receptor selectivity. Vice versa, LXR ligands behaved in non-selective manner, all activating at least PXR. CAR was selectively influenced by their ligands, while it also responded to several LXR ligands. Finally, although PXR was quite selectively activated or antagonized by its own ligands, it responded to several NRs ligands as well. Thus, using these reporter cell lines enabled us to precisely characterize the selectivity of pharmaceutical ligands for different nuclear receptors.

## Introduction

The superfamily of nuclear receptors (NRs) roofs the essential biological processes implying cell proliferation, differentiation, development and cell death, in both, normal and pathologic states (Aranda and Pascual, 2001; Evans and Mangelsdorf, 2014). NRs are transcription factors that comprise a modulatory A/B domain on N-terminus, a ‘hinge’ D domain, a ligand-binding domain (E domain), and a variable C-terminal F domain (Aranda and Pascual, 2001; Evans and Mangelsdorf, 2014; Weikum et al., 2018). Hydrophobic pocket of LBDs predispose NRs for response to specific ligands containing a range of endogenous hormonal or metabolic substances (bile acids, retinoids, steroid hormones, thyroid hormone, vitamin D), as well as a wide spectrum of ubiquitously present exogenous substances able to mimic or antagonize natural ligands (Weikum et al., 2018; Toporova and Balaguer, 2020). Among 48 members expressed in humans, 24 NRs perform as endogenous, ligand-activated transcription factors that in cooperation with coactivators/corepressors precisely regulate target gene expression. The remaining NRs called orphan receptors lack their regulatory ligands, so their transcriptional activity is alternatively regulated by post-translational events or expression of coregulatory proteins and eventually, by exogenous chemicals. In accordance to small sized, hydrophobic and lipid soluble character of endogenous molecules, there are plenty of suspected chemicals with pharmacological or industrial origin in the environment that potentially interact with NRs. In this regard, we took of interest the human farnesoid X receptor (FXR), the human liver X receptors (LXRα and LXRβ) and the human retinoic-acid-receptor-related orphan nuclear receptor γ (RORγ), serving as major actors of energy metabolism and circadian rhythm in organisms, transcriptionally regulating bile salt, cholesterol, fatty acid, and glucose homeostasis (Kalaany and Mangelsdorf, 2006; Evans and Mangelsdorf, 2014), as well as promiscuous xenobiotic receptors, the human constitutive androstane receptor (CAR) and the human pregnane X receptor (PXR), sensing to toxic by-products of endogenous metabolism, as well as exogenous chemicals arranging their elimination. As we have already done for other NRs (Seimandi et al., 2005; Grimaldi et al., 2015), we have established stable HG5LN-derived reporter cell lines in which FXR, LXRs, RORγ and CAR respective ligands induce luciferase activity. These cells stably express a chimeric protein containing the yeast transactivator GAL4 DBD fused to LBD regions of individual human NRs and contain luciferase reporter gene driven by recognition sequence of GAL4 pentamer in front of β-globin promoter. The GAL4-NR chimeric cell lines provide uniform cellular model differing just in receptor LBDs that eliminates background activities of endogenous receptors and allows accurate comparison of activity among receptors and their subtypes. As a proof of concept, we used these reporter cell lines to compare basal activity of FXR, LXRs, RORγ, CAR and PXR and finely characterize the selectivity of 22 commercially available pharmaceutical compounds. Regarding our results, we were able to confirm specific agonist/antagonist for nuclear receptors, reveal their selectivity or contrary, their promiscuity. We clearly demonstrated that *in vitro* cell-based assay enables an easy and rapid identification of nuclear receptor specific ligands with potential therapeutic applications.

## Material and Methods

### Chemicals

All tested compounds were obtained from Tocris (Bristol, UK) and their chemical structures are showed in supplementary (**Figure S1**). Chemicals were dissolved and stored as 10^-2^ M stock solutions in dimethyl sulfoxide (DMSO), while the final DMSO concentration never exceeded 0.1% (v/v) of the culture medium during testing.

### Plasmids

The plasmid (GAL4RE)5-βGlob-Luc-SVNeo (Chen et al., 1995) contains luciferase gene driven by a pentamer of yeast activator GAL4 binding sites in front of β-globin promoter and a neomycin phosphotransferase gene under the control of SV40 promoter. To construct GAL4-NR chimeras, individual LBDs of human FXR (R193-Q486), LXRα (A187-E447), LXRβ (G199-E461), CAR (M76-S348), and RORγ (S97-K518) were amplified by PCR with primers containing XhoI and SacI restriction sites (FXR), XhoI and BamHI (LXRs, CAR, RORγ) and inserted in pSG5-GAL4(DBD)-puromycin plasmid. To construct GAL4-CAR (+ APYLT) (M76-S353), the five amino acids APYLT were inserted by overlap extension PCR between amino acids P270 and D271 of CAR.

### Cell Lines

HG5LN and HG5LN PXR cells have already been described (Delfosse et al., 2015). To characterize the specificity of chemicals for human FXR, LXRα, LXRβ, CAR and RORγ, we established in a similar manner HG5LN FXR, LXRα, LXRβ, CAR WT, CAR (APYLT) and RORγ reporter cell lines. HG5LN are HeLa cells stably transfected by the (GAL4RE)5-βGlob-Luc-SVNeo plasmid (Seimandi et al., 2005). These cells were stably transfected by pSG5-GAL4 (DBD)-NRs (LBD)-puromycin plasmid and selected in presence of 0.5 µg/ml puromycin. Three weeks after the initiation of the puromycin selection, maximal luciferase expression of resistant clones was measured in presence of 1 µM GW4064 (HG5LN FXR), 1 µM T0901317 (HG5LN LXRs), DMSO (HG5LN RORγ and CAR), 1 µM CITCO (HG5LN CAR + APYLT) and 0.3 µM luciferin for 24h. Two days later, minimal luminescence expression from the same individual clones was measured in presence of 1 µM DY268 (HG5LN FXR), 1 µM SR9238 (HG5LN LXRs), 10 µM SR2211 (HG5LN RORγ), 1 µM PK1195 (HG5LN CAR and CAR + APYLT), and 0.3 µM luciferin. From each HG5LN NR cells, 5 to 10 clones were chosen for their ligand inducibility of luciferase expression. We noted that the basal activity in absence of ligand strongly differed among NRs. FXR, LXRs, and CAR APYLT had the lowest basal activities, whereas CAR and RORγ had the highest. The most inducible clones were expanded and rechecked for inducibility and aliquots were frozen at different passages. One clone per receptor was maintained in culture and used for ligand screening. HG5LN cells were cultured in Dulbecco’s modified Eagle’s medium (DMEM): Nutrient Mixture F-12 (DMEM/F-12) containing phenol red and 1 g/l glucose and supplemented with 5% fetal bovine serum, 100 units/ml of penicillin, 100 µg/ml of streptomycin and 1 mg/ml geneticin, in 5% CO_2_ humidified atmosphere at 37°C. Stably transfected cell lines derived from HG5LN cells were cultured in the same culture medium supplemented with 0.5 µg/ml puromycin.

### Transactivation Experiments

Individual reporter cell lines were seeded at a density of 40,000 cells per well in 96-well white opaque tissue culture plates (Greiner CellStar) in Dulbecco’s Modified Eagle Medium: Nutrient Mixture F-12 (DMEM/F-12) without phenol red and 1 g/l glucose and supplemented with 5% stripped fetal bovine serum, 100 units/ml of penicillin, 100 µg/ml of streptomycin (test medium). Chemicals to be tested were added 24h later, and cells were incubated at 37°C for 16h. Experiments were performed in quadruplicates. At the end of the incubation period, culture medium was replaced with test medium containing 0.3 mM luciferin solution. Luciferase activity was measured for 2s in intact living cells after 10 min stabilization using a Micro Beta Wallac luminometer (PerkinElmer). EC_50_ values were calculated using GraphPad Prism (GraphPad Software Inc).

Chemicals tested for agonism on CAR WT and RORγ were incubated in presence of 1 µM PK11195 and 1 µM SR2211. Chemicals tested for antagonism on FXR, LXRs, and PXR were incubated in presence of 100 nM GW4064 (HG5LN FXR), 20 nM T0901317 (HG5LN LXRα), 100 nM T0901317 (HG5LN LXRβ) 200 nM SR12813 (HG5LN PXR). Chemicals tested for agonism on FXR, LXRs, CAR (+ APYLT), PXR, and antagonism on CAR and RORγ were tested alone, without addition of any other ligand.

### Data Analysis and Statistics

In the transactivation assay, each compound was tested at various concentrations in three independent experiments at least. For each experiment, tests were performed in quadruplicates for each concentration, and data are expressed by means values with standard deviations. Individual agonist dose–response curves, in the absence and presence of antagonist, were fitted using the sigmoid dose-response function of a graphics and statistics software program (GraphPad Prism, version 5.0). EC_50_ (effective concentration for half-maximal luciferase activity) and IC_50_ (half-maximal inhibitory concentration) values were calculated from equations used to fit the data in this graphic software. Transactivation data are presented as EC_50_ and IC_50_ values for each compound tested. To analyze significances, we compared individual compound treatments with controls using one-way analysis of variance (ANOVA) with the help of GraphPad Prism software.

## Results

### Basal and Maximal Activity of HG5LN GAL4-NRs Cells

**Figure 1:**
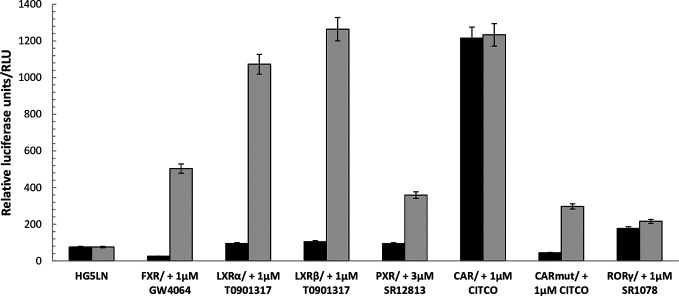
Baseline and maximal activities of the different Gal4 nuclear receptors. Basal and maximal luciferase expression of HG5LN and HG5LN GAL4-NRs cells were measured with and without addition of standard agonists. The expression of FXR and CAR APYLT induced a repression of luciferase expression, whereas the expression of CAR WT and RORγ increased it. The expression of LXRs and PXR had no effect on the basal transcription. Values represent the means and standard deviations of five different experiments.

To evaluate the FXR, LXRα, LXRβ, PXR, CAR, and RORγ specificity of pharmaceutical chemicals, we established HG5LN reporter cell lines expressing human FXR, LXRα, LXRβ, CAR, CAR (+ APYLT), and RORγ. Then, we tested the agonistic/antagonistic potential of 22 commercially available pharmaceutical compounds (**Figure S1**) on these cells and in the previously established PXR cell line (Delfosse et al., 2015). The 22 chemicals were first tested for non-specific modulation of luciferase expression on the HG5LN parental cell line, which contains the same reporter gene as HG5LN-GAL4-NRs cells, but lacks of Gal4-NRs. We found XY018, SPA70 and clotrimazole, as the most toxic representatives, while GW4064, DY268, GSK2033, WAY252623, and CITCO revealed vice versa, significant nonspecific activation (**Figure S2**). The basal (in absence of ligand) and the maximal (in presence of NR-specific full agonist) activities of HG5LN and the different HG5LN GAL4-NRs were measured. The maximal activity of FXR, LXRs, and PXR was obtained in the presence of 1 µM GW4064 (FXR), 1 µM T0901317 (LXRs), 3 µM SR12813 (PXR), respectively. The expression of FXR induced the slight repression of basal transcription, while both LXRs and PXR had no effect, or just slightly activated the basal transcription (**Figure 1**). Consequently, the baseline activities were normalized as percentage of maximal activity mediated by full agonists, as follows: 6% for FXR, 8% for LXRα, 9% for LXRβ, 19% for PXR (**Table 1**). On the contrary, the basal activity was increased by the expression of CAR and RORγ. Due to the inherent constitutive activity, we determined this activity equal to 100% as the specific agonists were not able to reveal their transactivation potential alone, but they were able to reverse the inhibition caused by antagonists/reverse agonists. To characterize easily CAR agonists, we used cell line expressing a variant of CAR (CAR + APYLT) with reduced basal (Auerbach et al., 2003). In this cell line, the baseline activity (11%) was normalized as percentage of maximal activity mediated by full agonist CITCO (**Table 1**) (Maglich et al., 2003). The difference in baseline activities of different NRs potentially results from receptor-specific recruitment of endogenous corepressors and/or coactivators presented in their apo forms. Subsequently, we tested all selected pharmaceutical compounds on our stable HG5LN GAL4-NRs reporter cell lines.

**Table 1:** Maximal activity and half maximal effective concentration (EC50) of the chemicals on FXR, LXRs, RORγ, CAR and PXR.

NR/Chemical		FXR		LXRα		LXRβ		RORγ (SR2211 1 mM)		CAR (APYLT)		PXR	
		% max act	EC50	% max act	EC50	% max act	EC50	% max act	EC50	% max act	EC50	% max act	EC50
	**DMSO**	6 ± 1	–	8 ± 1	–	9 ± 2	–	56 ± 5	–	11 ± 3	–	19 ± 2	–
**Ligands**													
**FXR**	GW4064*	100	41 ± 11	NE		NE		NE		NE		NE	
	**Feraxamine**	33	612 ± 188	NE		NE		NE		NE		NE	
	**DY268**	–	–	NE		NE		NE		NE		NE	
**LXR**	**T0901317**	–	–	100	9 ± 2	100	34 ± 8	20	–	NE		100	128 ± 12
	**GW3965**	–	–	98	176 ± 48	100	15 ± 6	NE		NE		88	1258 ± 67
	WAY252623**	NE		67	595 ± 68	72	321 ± 71	NE		NE		99	979 ± 91
	**SR9238**	NE		–	–	–	–	NE		NE		93	207 ± 13
	**SR9243**	NE		–	–	–	–	NE		NE		87	546 ± 89
	**GSK2033**	NE		–	–	–	–	NE		NE		92	505 ± 90
**RORγ**	SR1078**	NE		NE		NE		75	2091 ± 131	NE		NE	
	**SR0987**	NE		NE		NE		81	3186 ± 159	NE		75	209 ± 19.8
	**SR1001**	NE		NE		NE		–	–	NE		NE	
	SR2211**	NE		NE		NE		–	–	NE		NE	
	XY018*	NE		NE		NE		–	–	NE		NE	
**CAR**	CITCO*	NE		NE		NE		NE		100	380 ± 37.1	54	1293 ± 103
	**CINPA1**	NE		NE		NE		NE		NE		67	6853 ± 964
	**PK11195**	NE		NE		NE		NE		NE		80	726 ± 56
	**S07662**	NE		NE		NE		NE		NE		NE	
**PXR**	Clotrimazole*	NE		NE		NE		NE		23	> 10000	55	1278 ± 91
	**Dabrafenib**	NE		NE		NE		NE		NE		94	115 ± 16
	**SR12813**	NE		NE		NE		NE		NE		100	153 ± 52
	**SPA70**	NE		NE		NE		NE		NE		5	–

Values are the mean standard deviations from at least three separate experiments. Maximal activity of luciferase activity was determined at 10^-5^ M excepted for GW4064, WAY252623, SR1078, XY018, CITCO, and clotrimazole. For these compounds presenting nonspecific activation of the luciferase expression or toxicity, the maximal concentration tested was 10^-6^ M (GW4064, clotrimazole, CITCO and XY018)* or 3 10^-6^ M (WAY252623, SR1078, SR2211)**. EC_50_ was expressed in nM. NE, No effect.

### NRs Response to FXR Chemicals

**Figure 2:**
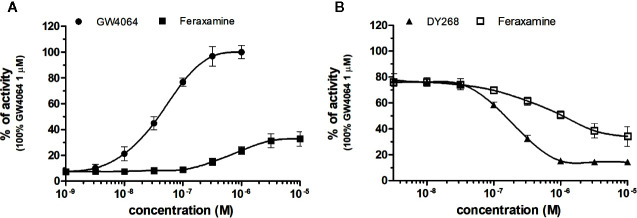
Dose–response curves of specific agonists **(A)** and antagonist **(B)** in HG5LN-hFXR cell line. Cells were incubated for 24 h with various concentrations of GW4064 **(A)**, Feraxamine **(A, B)** and DY268 **(B)**. The lines represent the best fit to the sigmoid dose–response curve equation of at least three separate experiments. The 100% value was obtained in the presence of 1 μM GW4064 and for respective antagonistic experiments 100 nM GW4064 corresponding to 80% of maximal activity was used. Results are expressed as percentages of luciferase activity measured in the wells. Values represent the means and standard deviations of three separate experiments.

FXR (NR1H4) was characterized as a nuclear receptor weakly activated by farnesol metabolites and by primary bile acids (Forman et al., 1995; Makishima et al., 1999). FXR also responds to lipophilic signaling molecules, including endocrine hormones, vitamins, xenobiotics, and dietary lipids. Three FXR ligands, two agonists (GW4064, feraxamine), and one antagonist (DY268) were tested, in HG5LN GAL4-FXR cells (**Figures 2A, B**). The full agonist GW4064 (Merk et al., 2019) acted as the most potent and efficacious FXR agonist with half-maximal effective concentration (EC_50_) equal to 41 nM (**Table 1**), which was in accordance with Zhang and co-workers (2015) obtained on HEK293T cells. Contrary to GW4064, feraxamine recently described as partial agonist (Merk et al., 2019) did not reach maximal FXR agonistic potential with an EC_50_ equal to 612 nM (**Figure 2A**, **Table 1**). Consistent with its partial agonist profile, feraxamine inhibited agonist-induced reporter activity of FXR chimeric receptor with corresponding concentration required to produce 50% of inhibition (IC_50_) calculated to 687 nM (**Figure 2B**, **Table 2**). As some full FXR agonists decreased the level of high-density lipoprotein (HDL), while increasing the level of low-density lipoprotein (LDL), leading finally to cardiovascular-related adverse effects, a partial agonists/antagonists could be promisingly less toxic than full agonists.

**Table 2:** Minimal activity and half maximal inhibitory concentration (IC50) of the chemicals on FXR, LXRs, RORγ, CAR, and PXR.

NR/Chemical		FXR (GW4064 100 nM)		LXRα (T0901317 20 nM)		LXRβ (T0901317 100 nM)		RORγ		CAR WT		PXR (SR12813 300 nM)		
		% min act	IC50	% min act	IC50	% min act	IC50	% min act	IC50	% min act	IC50	% min act	IC50	
	**DMSO**	80 ± 10	–	80 ± 5	–	80 ± 7	–	100 ± 5	–	100 ± 2	–	80 ± 9		–
**Ligands**														
**FXR**	GW4064*	–	–	NE		NE		NE		NE		NE		
	**Feraxamine**	33	687 ± 103	NE		NE		NE		NE		NE		
	**DY268**	14	148 ± 68	NE		NE		NE		NE		NE		
**LXR**	**T0901317**	28	5300 ± 234	–	–	–	–	20	1418 ± 177	40	4641 ± 597	–		–
	**GW3965**	25	5796 ± 313	–	–	–	–	NE		NE		–		–
	WAY252623**	NE		–	–	–	–	NE		NE		–		–
	**SR9238**	NE		2	26 ± 5	1	5 ± 1	NE		77	> 10000	–		–
	**SR9243**	NE		2	560 ± 99	2	59 ± 13	NE		65	8747 ± 3691	–		–
	**GSK2033**	NE		2	22 ± 6	2	10 ± 3	NE		21	556 ± 24	–		–
**RORγ**	SR1078**	NE		NE		NE		–	–	NE		NE		
	**SR0987**	NE		NE		NE		–	–	NE		–		–
	**SR1001**	NE		NE		NE		54	7385 ± 2111	NE		NE		
	SR2211**	NE		NE		NE		26	505 ± 238	NE		NE		
	XY018*	NE		NE		NE		58	1312 ± 329	NE		NE		
**CAR**	CITCO*	NE		NE		NE		NE		–	–	–		–
	**CINPA1**	NE		NE		NE		NE		19	251 ± 15	–		–
	**PK11195**	NE		NE		NE		NE		23	306 ± 21	–		–
	**S07662**	NE		NE		NE		NE		21	1056 ± 63	NE		
**PXR**	Clotrimazole*	NE		NE		NE		NE		64	2184 ± 181	–		–
	**Dabrafenib**	NE		NE		NE		NE		NE		–		–
	**SR12813**	NE		NE		NE		NE		NE		–		–
	**SPA70**	NE		NE		NE		NE		NE		5	278	± 35

Values are the mean standard deviations from at least three separate experiments. Maximal activity of luciferase activity was determined at 10^-5^ M excepted for GW4064, WAY252623, SR1078, XY018, CITCO and Clotrimazole. For these compounds presenting non specific activation of the luciferase expression or toxicity, the maximal concentration tested was 10^-6^ M (GW4064, clotrimazole, CITCO and XY018)* or 3 10^-6^ M (WAY252623, SR1078, SR2211)**. IC50 was expressed in nM. NE, No effect.

Finally, DY268, trisubstituted-pyrazol carboxamide (IC_50_ of 468.5 nM in FXR cell-based antagonistic assays published by Yu et al., 2014), behaved as a full FXR antagonist, able to inhibit FXR activity with an IC_50_ of 148 nM (**Figure 2B**, **Table 2**). Interestingly, all these compounds showed full FXR selectivity, as they did not activated/inhibited the other tested NRs (**Tables 1** and **2**). FXR ligand specificity is the important observation, considering the beneficial effects of FXR ligands in cholestasis and hypercholesterolemia (Jonker et al., 2012; Amano et al., 2018; Keitel et al., 2019).

### NRs Response to LXRs Chemicals

**Figure 3:**
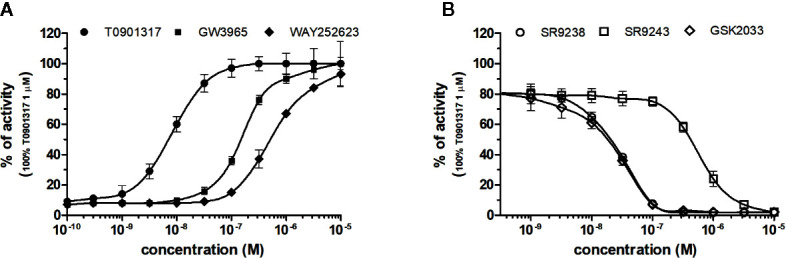
Dose–response curves of specific agonists **(A)** and antagonists **(B)** in HG5LN- LXRα cell line. Cells were incubated for 24 h with various concentrations of agonists T0901317, GW3965 and WAY252623 **(A)**, as well as antagonists SR9238, SR9243, and GSK2033 **(B)**. The lines represent the best fit to the sigmoid dose–response curve equation of three separate experiments. The 100% value was obtained in the presence of 1 μM T0901317 and for respective antagonistic experiments 20 nM T0901317 corresponding to 80% of maximal activity was used. Results are expressed as percentages of luciferase activity measured in the wells. Values represent the means and standard deviations of three separate experiments.

**Figure 4:**
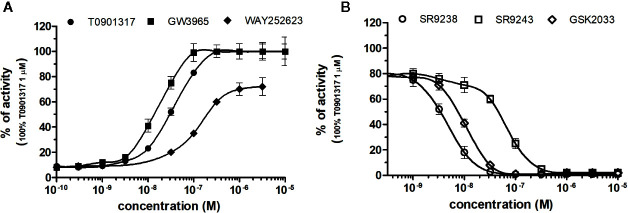
Dose–response curves of specific agonists **(A)** and antagonists **(B)** in HG5LN- LXRβ cell line. Cells were incubated for 24 h with various concentrations of agonists T0901317, GW3965 and WAY252623 **(A)**, as well as antagonists SR9238, SR9243, and GSK2033 **(B)**. The lines represent the best fit to the sigmoid dose–response curve equation of three separate experiments. The 100% value was obtained in the presence of 1 μM T0901317 and for respective antagonistic experiments 100 nM T0901317 corresponding to 80% of maximal activity was used. Results are expressed as percentages of luciferase activity measured in the wells. Values represent the means and standard deviations of three separate experiments.

LXRα and LXRβ (also known as NR1H3 and NR1H2) originally considered to be orphan nuclear receptors, were “adopted” following the discovery that metabolites of cholesterol bind to and activate these receptors at physiological concentrations and induce the expression of genes involved in lipid metabolism (Willy, 1995; Gill et al., 2008). Six LXR ligands, three agonists (T0901317, GW3965 and WAY252623) and three antagonists (SR9238, SR9243 and GSK2033) were tested in HG5LN GAL4-LXRs cells (**Figures 3** and **4**). T0901317 was settled as reference 100% value of activity (1 µM) for both, LXRα and LXRβ. T0901317 revealed mild subtype specificity favoring α subtype with EC_50_ values corresponding to 9 nM (LXRα) and 34 nM (LXRβ) (**Figures 3A** and **4A**, **Table 1**). Indeed, T0901317 is the agonist for multiple targets, which possesses EC_50_ values of 20 nM for LXRα and lesser, micromolar extent for LXRβ, respectively, in HEK293 cells (Schultz et al., 2000; Kanno et al., 2013). GW3965 was also a potent full agonist that revealed 12-fold LXRβ specificity (**Figures 3A** and **4A**, **Table 1**). EC_50_ values corresponded to 176 nM (LXRα) and 15 nM (LXRβ). WAY252623 activated LXRs with lower potency and almost no subtype specificity (EC_50_ values of 595 and 321 nM for LXRα and LXRβ (**Figures 3A** and **4A**, **Table 1**). In accordance with our results, tertiary amine GW3965, activated a luciferase reporter gene containing LXR response elements in HEK293 cells expressing LXRα or LXRβ, while revealed nanomolar LXRβ agonistic potency (Peng et al., 2011).

LXRs antagonists were tested in presence of T0901317 (20 and 100 nM for LXRα and LXRβ respectively). The IC_50_ of SR9238 was 26 nM for LXRα and 5 nM for LXRβ showing high LXR potency and 5-fold LXR β-subtype specificity to the detriment of α subtype (**Table 2**). SR9243 antagonized T0901317-induced luciferase expression in both, HG5LN GAL4-LXRα and LXRβ cell lines with higher potency for LXRβ (IC_50_ of 560 and 59 nM for LXRα and LXRβ, respectively) (**Figures 3B** and **4B**, **Table 2**). GSK2033 resulted as well, in high potent inhibition of LXRs with appropriate IC_50_ values corresponding to 22 nM for LXRα and 10 nM for β subtype (**Figures 3B** and **4B**, **Table 2**). Remarkably, these three chemicals repressed the basal activities of LXRα and LXRβ that clearly correspond to their inverse agonistic potential, which is in accordance with previous studies (Griffett et al., 2013; Flaveny et al., 2015).

**Figure 5:**
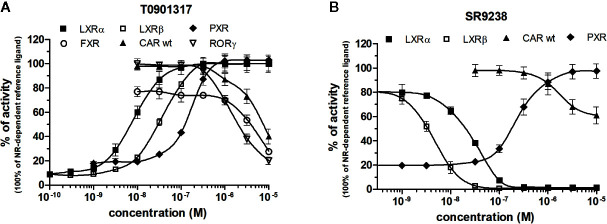
Dose–response curves of the most promiscuous chemicals T0901317 **(A)** and SR9238 **(B)** in HG5LN-NR cell lines. Cells were incubated for 24 h with ligands ranging in 0.1 nM - 10 μM. The 100% value for LXRs was obtained in the presence of 1 μM T0901317. The 100% value for PXR was obtained in the presence of 3 μM SR12813. The 100% value for FXR was obtained in the presence of 1 μM GW4064. The 100% value for CAR and RORγ was settled as receptor constitutive activity without addition of ligand. For FXR antagonistic experiments, 100 nM GW4064 corresponding to 80% of maximal activity was used. For LXRα antagonistic experiments, 20 nM T0901317 corresponding to 80% of maximal activity was used. For LXRβ, antagonistic experiments, 100 nM T0901317 corresponding to 80% of maximal activity was used. The lines represent the best fit to the sigmoid dose–response curve equation of three separate experiments. Results are expressed as percentages of luciferase activity measured in the wells. Values represent the means and standard deviations of three separate experiments.

LXRs ligands appeared to be remarkably nonspecific among the tested molecules. Previous studies showed that T0901317 activated/repressed several tested receptors (Houck et al., 2004; Xue et al., 2007; Kanno et al., 2013). Indeed, it appeared to be the least selective compound (**Figure 5A**), since apart from its agonistic activity on LXRs was able to dose-dependently activate PXR (EC_50_ of 128 nM), inhibit agonist-induced reporter activity of FXR (IC_50_ of 5300 nM) and even repress constitutive activities of CAR (IC_50_ of 4641 nM) and RORγ (IC_50_ of 1418 nM) (**Figure 5A**, **Tables 1** and **2**). With lower extent, GW3965 showed additional agonistic properties against PXR and even antagonized FXR (**Tables 1** and **2**). LXR antagonists, SR9238, SR9243 and GSK2033 behaved in the similar manner, as they did not only efficiently inhibited T0901317-induced LXR activity under receptor basal level, but all of them, with SR9238 on the forefront, activated PXR and slightly repressed constitutive activity of CAR WT (**Figure 5B**, **Tables 1** and **2**). We also observed substantial receptor non-specificity of GSK2033, activating PXR with EC_50_ equal to 505 nM on the one hand (**Table 1**), and repressing constitutive activity of CAR with IC_50_ of 556 nM on the other (**Table 2**). In accordance with our observations, recently published hypothesis believes in promiscuity of GSK2033, as it potentially targets several receptors (Griffett and Burris, 2016).

### NRs Response to RORγ Chemicals

**Figure 6:**
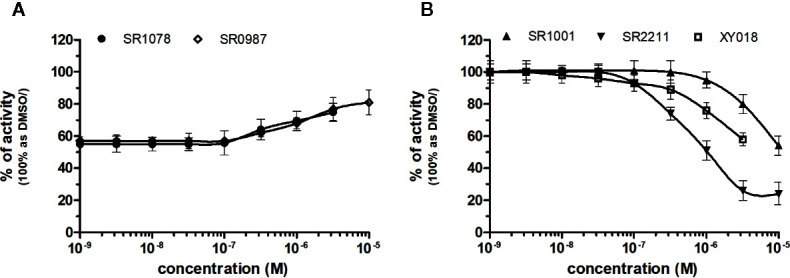
Dose–response curves of specific agonists **(A)** and antagonists **(B)** in HG5LN-RORγ cell line. Cells were incubated for 24 h with various concentrations of agonists SR1078 and SR0987 in presence of antagonist SR2211 at 1 µM repressing the high constitutive activity of receptor **(A)**. As well, we incubated cells with antagonists **(B)** SR1001, SR2211, and XY018. The lines represent the best fit to the sigmoid dose–response curve equation of three separate experiments. The inherent high basal activity of RORγ was settled as 100%. Results are expressed as percentages of luciferase activity measured in the wells. Values represent the means and standard deviations of three separate experiments.

RORγ (NR1F3) binds and is preferentially modulated by oxysterol derivatives and not by retinoic acid (Fauber and Magnuson, 2014). SR1078 and SR0987 were identified as chemicals that induced the expression of the ROR target genes, where SR1078 showed concentration dependent induction of reporter gene expression with EC_50_ of 800 nM, while its synthetic analogue SR0987 demonstrated better efficacy in full-length RORγt transfected cells (The correct citation is: Rene et al., 2014; Chang et al., 2016). By applying referred agonists, SR1078 and SR0987 in combination with inverse agonist SR2211 (1 μM) on our chimeric reporter cell line containing just LBD of RORγ, both SR1078 and SR0987 revealed agonistic properties with corresponding EC_50_ of 2,091 and 3,186 nM (**Figure 6A**, **Table 1**).

Due to the constitutive activity of RORγ, the antagonists SR2211, SR1001 and XY018 inhibited the constitutive activity of RORγ with IC_50_s of 505, 1,312, and 7,385 nM for SR2211, XY018 and SR1001, respectively (**Figure 6B**, **Table 2**). It is noteworthy, that administration of the inverse RORγ agonists has proven pharmaceutical potential, as SR1001 significantly reduced diabetes incidence and insulitis in a type 1 diabetes model mice (Solt et al., 2015) and SR2211 has been reported to inhibit inflammation in a collagen-induced arthritis mouse model (Xue et al., 2016). RORγ ligands appeared relatively specific among tested molecules, as only SR0987 activated PXR and CAR in the submicromolar concentrations (**Tables 1** and **2**).

### NRs Response to CAR Chemicals

**Figure 7:**
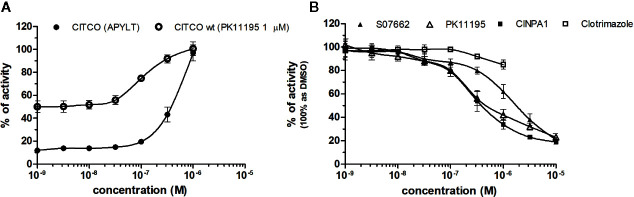
Dose–response curves of CITCO **(A)** and CAR antagonists **(B)** in HG5LN CAR cell lines. HG5LN CAR and HG5LN CAR (+APYLT) cells were incubated for 24 h with various concentrations of CITCO **(A)**. Response of CITCO was tested in presence 1 μM of PK1195 used for reversion of CAR WT constitutive activity, as well as alone in CAR APYLT mutant with low basal activity. HG5LN CAR cells were incubated for 24 h with various concentrations of inverse agonists S07662, CINPA1, and PK11195 **(B)**. The lines represent the best fit to the sigmoid dose–response curve equation of three separate experiments. The 100% value was settled as receptor constitutive activity without addition of ligand. Results are expressed as percentages of luciferase activity measured in the wells. Values represent the means and standard deviations of three separate experiments.

CAR (NR1I3) similarly to PXR is a key regulator of metabolism and play a major role in detoxification. The constitutive activity of CAR and its constitutive nuclear localization in cancer cell lines did not allow to test directly chemicals with agonistic activity. To reveal the agonistic activity of 22 chemicals in our HG5LN GAL4-hCAR cell line, we tested their ability to reverse the inhibitory effect of PK11195 (1 μM), one of the most potent CAR antagonist. In these conditions, only the well know CAR agonist CITCO (EC_50_ of 49 nM in the CAR/SRC-1 FRET assay published by Maglich et al., 2003) showed strong transactivation potential with EC_50_ of 253 nM (data not shown). As ligand-dependency of CAR is increased in a CAR variant having an insertion of five amino acids (APYLT) residues into the ligand binding domain (Auerbach et al., 2003), we generated a HG5LN cells expressing this CAR variant (HG5LN CAR + APYLT). In accordance with our expectations, CITCO fully activated our chimeric CAR+APYLT cell line with EC_50_ equal to 380 nM (**Figure 7A**, **Table 1**). It is noteworthy, that the insertion of five amino acids with consequent decrease of receptor constitutive activity, facilitates the screening of CAR agonists.

To reveal the antagonistic activity of 22 chemicals, we tested their ability to inhibit effect the basal activity in our HG5LN GAL4-CAR WT cell line. In this regard, the potency of the most efficacious reference compounds S07662, CINPA1 and PK11195 was sought in dose-response. These experiments resulted in significant suppression of receptor constitutive activity with IC_50_s of 251, 306 and 1056 nM for CINPA1, PK11195 and S07662, respectively (**Figure 7B**, **Table 2**). Except S07662, all of the CAR ligands behaved as PXR agonists, while CITCO being the most potent with an EC_50_ for PXR of 1.3 µM, as it was recently published (Anderson et al., 2011; Lin et al., 2020).

### NRs Response to PXR Chemicals

PXR, another xenobiotic sensor among nuclear receptors, is commonly known to be activated by a very structurally diverse array of endogenous and exogenous molecules referring its expansive (~1,300 Å3), hydrophobic, roughly spherical ligand binding pocket with the flexibility to accommodate large molecules. This fact was fully supported by our experiments, where several tested pharmaceutics unveiled agonistic properties for PXR.

**Figure 8:**
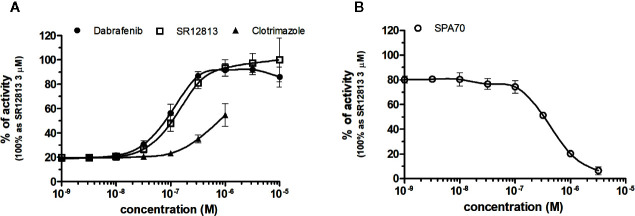
Dose–response curves of specific agonists **(A)** and antagonist **(B)** in HG5LN-PXR cell line. Cells were incubated for 24 h with various concentrations of agonists SR12813 and Dabrafenib **(A)** as well as antagonist SPA70 **(B)**. The lines represent the best fit to the sigmoid dose–response curve equation of three separate experiments. The 100% value was obtained in the presence of 3 μM SR12813 and for respective antagonistic experiments 300 nM SR12813 corresponding to 80% of maximal activity was used. Results are expressed as percentages of luciferase activity measured in the wells. Values represent the means and standard deviations of three separate experiments.

SR12813 referred as high affinity ligand for PXR (EC_50_ of 0.44 μM in hPXR LBD assay performed by Shukla et al., 2011) showed strong PXR selectivity with corresponding EC_50_ of 153 nM in our experiments. Dabrafenib (Creusot et al., manuscript in preparation) was little more potent with an EC_50_ of 115 nM, while less potency revealed clotrimazole with EC_50_ of 1,278 nM (**Figure 8A**, **Table 1**). Plenty of drugs bind to and activate PXR while upregulating drug-metabolizing enzymes, decrease drug efficacy and increase resistance. Taking together, PXR antagonists could have therapeutic exploitation. Indeed, SPA70 was reviewed as potent and selective PXR antagonist (IC_50_ of 510 nM in the cell-based hPXR antagonistic assay), while inhibited PXR activities in human hepatocytes or humanized mouse models and enhanced the chemosensitivity of cancer cells, consistent with the role of PXR in drug resistance (Lin et al., 2017). In our hands, SPA70 was able to inhibit agonist-induced reporter activity with an IC_50_ of 278 nM and displayed a strong selectivity for PXR (**Figure 8B**, **Table 2**).

## Discussion

Selective agonistic and antagonistic ligands are the powerful tools for study the functions of nuclear receptors. Although several synthetic NR ligands are commercially available, their selectivity among nuclear receptors has not always been thoroughly evaluated. For this purpose we developed cellular assay for identification and characterization of FXR, LXRs, RORγ, PXR, and CAR ligands. We employed GAL4-NR chimeric receptors, because this assay format eliminates the background activities of endogenous receptors and allows the comparison of different NRs activities with the same reporter gene.

Using these cells, we observed that the expression of individual FXR, LXRs, RORγ, PXR, and CAR differentially modulated the reporter gene basal activities, thus providing important information of recruitment the HeLa-specific coregulators by NRs. Indeed, the baseline activities were different in the HG5LN FXR, LXRs, RORγ, PXR, and CAR cell lines. Specifically, the expression of FXR and CAR + APYLT induced a repression of luciferase expression, whereas the expression of CAR WT and RORγ was increased. It is noteworthy that RORγ and CAR WT adopt an active conformation in their apo form and thus are constitutively activated in absence of ligand (Jin et al., 2010; Lee et al., 2017; Pham et al., 2019). Contrary, the expression of LXRs and PXR had no effect on the basal transcription in our experimental set-up (**Figure 1**).

Then we tested the selectivity of chemicals for their receptors. The full agonist GW4064, the partial agonist feraxamine and the full antagonist DY268 are perfectly selective for FXR, as they did not modulate the activity of the other tested NRs. In a similar manner, the full agonist SR12813 and the full antagonist SPA70 are perfectly selective for PXR, as they did not modulate the activity of the other NRs. It is remarkable that Lin et al. (2017) were able to discover a selective antagonist with good affinity for PXR, since it was believed for a long time that this receptor could not be antagonized due to its ligand promiscuity (Chai et al., 2019). RORγ agonist and antagonists are also relatively selective. Contrary, LXR agonists and antagonists are much less selective, as they modulated the activities of PXR and CAR. Clearly, further efforts are necessary, to develop the chemicals with better selectivity for these receptors. In a similar manner, CAR ligands except S07662 activated PXR. As PXR and CAR are known to regulate an overlapping set of genes (Banerjee et al., 2015; Buchman et al., 2018; Chai et al., 2019), it is important to develop more selective CAR ligands.

Among the different NRs tested in our study, PXR is the most promiscuous. Higher promiscuity of PXR compared to FXR, LXRs and RORγ is certainly linked to its major roles in the modulation of enzymes involved in the biotransformation, metabolism and elimination of xenobiotics and endobiotics (Buchman et al., 2018). Moreover, PXR is notable for having a flexible, dynamic and hydrophobic LBD that can accommodate a wide range of ligands with diverse structural and physicochemical properties. Crystallographic studies have revealed that PXR possesses a large LBP (>1150 Å3) that can accommodate compounds with larger volumes than that of classical NR ligands, and accordingly, several loops of the LBD confer a high plasticity allowing the receptor to adopt different shapes due to the bound ligands. In this context regarding the biological involvement in xenobiotic metabolism, together with not so much smaller and less flexible size of CAR ligand-binding cavity of (~675 Å3), we could possibly expect comparable promiscuity of CAR with PXR.

Finally, these reporter cell lines that we developed allowed us to characterize the potency, efficacy and selectivity of 22 synthetic NRs ligands in a standardized, high-throughput screening technique using 96-well plates. In addition, the HG5LN parental cell line, which expresses only the GAL4-driven reporter gene, was used to ascertain that the activation of the HG5LN GAL4-NR reporter gene was mediated by NRs. We conclude that these reporter cell lines in addition with previously developed HG5LN GAL4-PPARs cells (Seimandi et al., 2005) allow specific and sensitive measurement of NR ligand activities and are a high-throughput, cell-based screening tool for identifying and characterizing ligands for the main NRs, the targets of pharmaceuticals. Following work will consist of study the activity and selectivity of these ligands *in vivo*, in order to reveal their bioavailability and metabolism.

## Data Availability Statement

The raw data supporting the conclusions of this article will be made available by the authors, without undue reservation, to any qualified researcher.

## Author Contributions

LT and MG performed experiment. LT and AB established reporter cell lines. LT and PB wrote the article. PB supervised the work. All authors contributed to the article and approved the submitted version.

## Funding

This work was supported by ANSES (project TOXCHEM 2018/1/095), Environment-cancer (project SYNERPXR and SYNERPXR 2), PNRPE (PESTR and SYNEPEST), and ANR (Project SYNERACT R18083FF) grants.

## Conflict of Interest

The authors declare that the research was conducted in the absence of any commercial or financial relationships that could be construed as a potential conflict of interest.

## Supplementary Material

Figure S1 Chemical structure of tested pharmaceutical compounds.

Figure S2 Nonspecific activation/inhibition of luciferase expression by the tested pharmaceuticals in the parental cell line HG5LN.

## References

[B1] Amano Y. Yamakawa H. Yonemori K. Shimada M. Tozawa R. (2018). Farnesoid X receptor antagonist exacerbates dyslipidemia in mice. Pharmacol. Rep. 70 (1), 172–177. 10.1016/j.pharep.2017.07.010 29367104

[B2] Anderson L. E. Dring A. M. Hamel L. D. Stoner M. A. (2011). Modulation of constitutive androstane receptor (CAR) and pregnane X receptor (PXR) by 6-arylpyrrolo[2,1-d][1,5]benzothiazepine derivatives, ligands of peripheral benzodiazepine receptor (PBR).Toxicol. Lett.er 202 (2), 148–154. 10.1016/j.toxlet.2011.02.004 PMC3086002 21315811

[B3] Aranda A. Pascual A. (2001). Nuclear Hormone Receptors and Gene Expression. Physiol. Rev. 81 (3), 1269–1304. 10.1152/physrev.2001.81.3.1269 11427696

[B4] Auerbach S. S. Ramsden R. Stoner M. A. Verlinde C. Hassett C. Omiecinski C. J. (2003). Alternatively spliced isoforms of the human constitutive androstane receptor. Nucleic Acids Res. 31 (12), 3194–3207. 10.1093/nar/gkg419 12799447 PMC162252

[B5] Banerjee M. Robbins D. Chen T. (2015). Targeting xenobiotic receptors PXR and CAR in human diseases. Drug Discovery Today 20 (5), 618–628. 10.1016/j.drudis.2014.11.011 25463033 PMC4433851

[B6] Buchman C. D. Chai S. C. Chen T. (2018). A current structural perspective on PXR and CAR in drug metabolism. Expert Opin. Drug Metab. Toxicol. 14 (6), 635–647. 10.1080/17425255.2018.1476488 29757018 PMC6002932

[B7] Chai S. C. Wright W. C. Chen T. (2019). Strategies for developing pregnane X receptor antagonists: Implications from metabolism to cancer. Med. Res. Rev. 24 (3), 906–915. 10.1002/med.21648 PMC7166136 31782213

[B8] Chang M. R. Dharmarajan V. Doebelin C. Garcia-Ordonez R. D. Novick S. J. Kuruvilla D. S. (2016). Synthetic RORγt Agonists Enhance Protective Immunity. ACS Chem. Biol. 11 (4), 1012–1018. 10.1021/acschembio.5b00899 26785144 PMC5178133

[B9] Chen J. Y. Penco S. Ostrowski J. Balaguer P. Pons M. Starrett J. E. (1995). RAR-specific agonist/antagonists which dissociate transactivation and AP1 transrepression inhibit an chorage-independent cell proliferation. EMBO J. 14 (6), 1187–1197. 10.1002/j.1460-2075.1995.tb07102.x 7720709 PMC398196

[B10] Delfosse V. Dendele B. Huet T. Grimaldi M. Boulahtouf A. Gerbal-Chaloin S. (2015). Synergistic activation of human pregnane X receptor by binary cocktails of pharmaceutical and environmental compounds. Nat. Commun. 6, 8089. 10.1038/ncomms9089 26333997 PMC4569708

[B11] Evans R. M. Mangelsdorf D. J. (2014). Nuclear Receptors, RXR, and the Big Bang. Cell 157 (1), 255–266. 10.1016/j.cell.2014.03.012 24679540 PMC4029515

[B12] Fauber B. P. Magnuson S. (2014). Modulators of the Nuclear Receptor Retinoic Acid Receptor-Related Orphan Receptor-γ (RORγ or RORc) J. Med. Chem. 57 (14), 5871–5892. 10.1021/jm401901d 24502334

[B13] Flaveny C. A. Griffett K. El-Gendy B. E. D. M. Kazantzis M. Sengupta M. Amelio A. L. (2015). Broad Anti-tumor Activity of a Small Molecule that Selectively Targets the Warburg Effect and Lipogenesis. Cancer Cell. 28 (1), 42–56. 10.1016/j.ccell.2015.05.007 26120082 PMC4965273

[B14] Forman B. M. Goode E. Chen J. Oro A. E. Bradley D. J. Perlmann T. (1995). Identification of a nuclear receptor that is activated by farnesol metabolites. Cell 81 (5), 687–693. 10.1016/0092-8674(95)90530-8 7774010

[B15] Gill S. Chow R. Brown A. J. (2008). Sterol regulators of cholesterol homeostasis and beyond: the oxysterol hypothesis revisited and revised. Prog. Lipid Res. 47 (6), 391–404. 10.1016/j.plipres.2008.04.002 18502209

[B16] Griffett K. Burris T. P. (2016). Promiscuous activity of the LXR antagonist GSK2033 in a mouse model of fatty liver disease. Biochem. Biophys. Res. Commun. 479 (3), 424–428. 10.1016/j.bbrc.2016.09.036 27680310 PMC5087326

[B17] Griffett K. Solt L. A. El-Gendy B. E. Kamenecka T. M. Burris T. P. (2013). A liver-selective LXR inverse agonist that suppresses hepatic steatosis. ACS Chem. Biol. 8 (3), 559–567. 10.1021/cb300541g 23237488

[B18] Grimaldi M. Boulahtouf A. Delfosse V. Thouennon E. Bourguet W. Balaguer P. (2015). Reporter Cell Lines for the Characterization of the Interactions between Human Nuclear Receptors and Endocrine Disruptors. Front. Endocrinol. (Lausanne) 6:62. 10.3389/fendo.2015.00062 26029163 PMC4426785

[B19] Houck K. A. Borchert K. M. Hepler C. D. Thomas J. S. Bramlett K. S. Michael L. F. (2004). T0901317 is a dual LXR/FXR agonist. Mol. Gen. Metab. 83 (1-2), 184–187. 10.1016/j.ymgme.2004.07.007 15464433

[B20] Jin L. Martynowski D. Zheng S. Wada T. Xie W. Li Y. (2010). Structural basis for hydroxycholesterols as natural ligands of orphan nuclear receptor RORgamma. Mol. Endocrinol. 24 (5), 923–929. 10.1210/me.2009-0507 20203100 PMC2870936

[B21] Jonker J. W. Liddle C. Downes M. (2012). FXR and PXR: potential therapeutic targets in cholestasis. J. Steroid Biochem. Mol. Biol. 130 (3-5), 147–158. 10.1016/j.jsbmb.2011.06.012 21801835 PMC4750880

[B22] Kalaany N. Y. Mangelsdorf D. J. (2006). LXRS AND FXR: The Yin and Yang of Cholesterol and Fat Metabolism. Annu. Rev. Physiol. 68, 159–191. 10.1146/annurev.physiol.68.033104.152158 16460270

[B23] Kanno Y. Tanuma N. Takahashi A. Inouye Y. (2013). T0901317, a potent LXR agonist, is an inverse agonist of CAR. J. Toxicol. Sci. 38 (3), 309–315. 10.2131/jts.38.309 23665929

[B24] Keitel V. Dröge C. Häussinger D. (2019). Targeting FXR in Cholestasis. Handb. Exp. Pharmacol. 256, 299–324. 10.1007/164_2019_231 31201556

[B25] Lee K. You H. Choi J. No K. T. (2017). Development of pharmacophore-based classification model for activators of constitutive androstane receptor. Drug Metab. Pharmacokinet. 32 (3), 172–178. 10.1016/j.dmpk.2016.11.005 28366619

[B26] Lin W. Wang Y. M. Chai S. C. Lv L. Zheng J. Wu J. (2017). SPA70 is a potent antagonist of human pregnane X receptor. Nat. Commun. 8 (1), 741. 10.1038/s41467-017-00780-5 28963450 PMC5622171

[B27] Lin W. Bwayi M. Wu J. Li Y. Chai S. C. Huber A. D. (2020). CITCO directly binds to and activates human Pregnane X Receptor. Mol. Pharmacol. 97 (3), 180–190. 10.1124/mol.119.118513 31882411 PMC6978709

[B28] Maglich J. M. Parks D. J. Moore L. B. Collins J. L. Goodwin B. Billin A. N. (2003). Identification of a novel human constitutive androstane receptor (CAR) agonist and its use in the identification of CAR target genes. J. Biol. Chem. 278 (19), 17277–17283. 10.1074/jbc.M300138200 12611900

[B29] Makishima M. Okamoto A. Y. Repa J. J. Tu H. Learned R. M. Luk A. (1999). Identification of a nuclear receptor for bile acids. Science 21;284 (5418), 1362–1365. 10.1126/science.284.5418.1362 10334992

[B30] Merk D. Sreeramulu S. Kudlinzki D. Saxena K. Linhard V. Gande S. L. (2019). Molecular tuning of farnesoid X receptor partial agonism. Nat. Commun. 10 (1), 2915. 10.1038/s41467-019-10853-2 31266946 PMC6606567

[B31] Peng D. Hiipakka R. A. Xie J. T. Dai Q. Kokontis J. M. Reardon C. A. (2011). A novel potent synthetic steroidal liver X receptor agonist lowers plasma cholesterol and triglycerides and reduces atherosclerosis in LDLR(-/-) mice. Br. J. Pharmacol. 162 (8), 1792–1804. 10.1111/j.1476-5381.2011.01202.x 21232031 PMC3081122

[B32] Pham B. Arons A. B. Vincent J. G. Fernandez E. J. Shen T. (2019). Regulatory Mechanics of Constitutive Androstane Receptors: Basal and Ligand-Directed Actions. J. Chem. Inf. Model. 59 (12), 5174–5182. 10.1021/acs.jcim.9b00695 31714771

[B33] Rene O. Fauber B. P. de Boenig G. L. Burton B. Eidenschenk C. Everett C. (2014). Minor Structural Change to Tertiary SulfonamideRORc Ligands Led to Opposite Mechanisms of Action. ACS Med. Chem. Lett. 6 (3), 276–281. 10.1021/ml500420y 25815138 PMC4360161

[B34] Schultz J. R. Tu H. Luk A. Repa J. J. Medina J. C. Li L. (2000). Role of LXRs in control of lipogenesis. Genes Dev. 14, 2831– 2838. 10.1101/gad.850400 11090131 PMC317060

[B35] Seimandi M. Lemaire G. Pillon A. Perrin A. Carlavan I. Voegel J. J. (2005). Differential responses of PPARalpha, PPARdelta, and PPARgamma reporter cell lines to selective PPAR synthetic ligands. Anal. Biochem. 344 (1), 8–15. 10.1016/j.ab.2005.06.010 16038868

[B36] Shukla S. J. Sakamuru S. Huang R. Moeller T. A. Shinn P. Vanleer D. (2011). Identification of clinically used drugs that activate pregnane X receptors. Drug Metab. Dispos.: Biol. Fate Chem. 39 (1), 151–159. 10.1124/dmd.110.035105 20966043 PMC3014269

[B37] Solt L. A. Banerjee S. Campbell S. Kamenecka T. M. Burris T. P. (2015). ROR inverse agonist suppresses insulitis and prevents hyperglycemia in a mouse model of type 1 diabetes. Endocrinology 156 (3), 869–881. 10.1210/en.2014-1677 25560829 PMC4330305

[B38] Toporova L. Balaguer P. (2020). Nuclear receptors are the major targets of endocrine disrupting chemicals. Mol. Cell. Endocrinol. 502:110665. 10.1016/j.mce.2019.110665 31760044

[B39] Weikum E. R. Liu X. Ortlund E. A. (2018). The nuclear receptor superfamily: A structural perspective. Protein Sci. 27 (11), 1876–1892. 10.1002/pro.3496 30109749 PMC6201731

[B40] Willy P. J. (1995). LXR, a nuclear receptor that defines a distinct retinoid response pathway. Genes Dev. 9 (9), 1033–1045. 10.1101/gad.9.9.1033 7744246

[B41] Xue Y. Chao E. Zuercher W. J. Willson T. M. Collins J. L. Redinbo M. R. (2007). Crystal structure of the PXR-T1317 complex provides a scaffold to examine the potential for receptor antagonism. Bioorg. Med. Chem. 15 (5), 2156–2166. 10.1016/j.bmc.2006.12.026 17215127 PMC1839856

[B42] Xue X. Soroosh P. De Leon-Tabaldo A. Luna-Roman R. Sablad M. Rozenkrants N. (2016). Pharmacologic modulation of RORγt translates to efficacy in preclinical and translational models of psoriasis and inflammatory arthritis. Sci. Rep. 6:37977. 10.1038/srep37977 27905482 PMC5131364

[B43] Yu D. D. Lin W. Forman B. M. Chen T. (2014). Identification of trisubstituted-pyrazol carboxamide analogs as novel and potentant agonists of farnesoid X receptor. Bioorg. Med. Chem. 22 (11), 2919–2938. 10.1016/j.bmc.2014.04.014 24775917 PMC4147378

[B44] Zhang S. Pan X. Jeong H. (2015). GW4064, an agonist of farnesoid X receptor, represses CYP3A4 expression in human hepatocytes by inducing small heterodimer partner expression. Drug Metab. Dispos. 43 (5), 743–748. 10.1124/dmd.114.062836 25725071 PMC4407707

