# Supplementary figures and images for: Assessing the Selectivity of FXR, LXRs, CAR, and RORγ Pharmaceutical Ligands With Reporter Cell Lines
Source: Front Pharmacol. 2020 Jul 24;11:1122. doi: 10.3389/fphar.2020.01122 (PMC7394005; doi:10.3389/fphar.2020.01122)

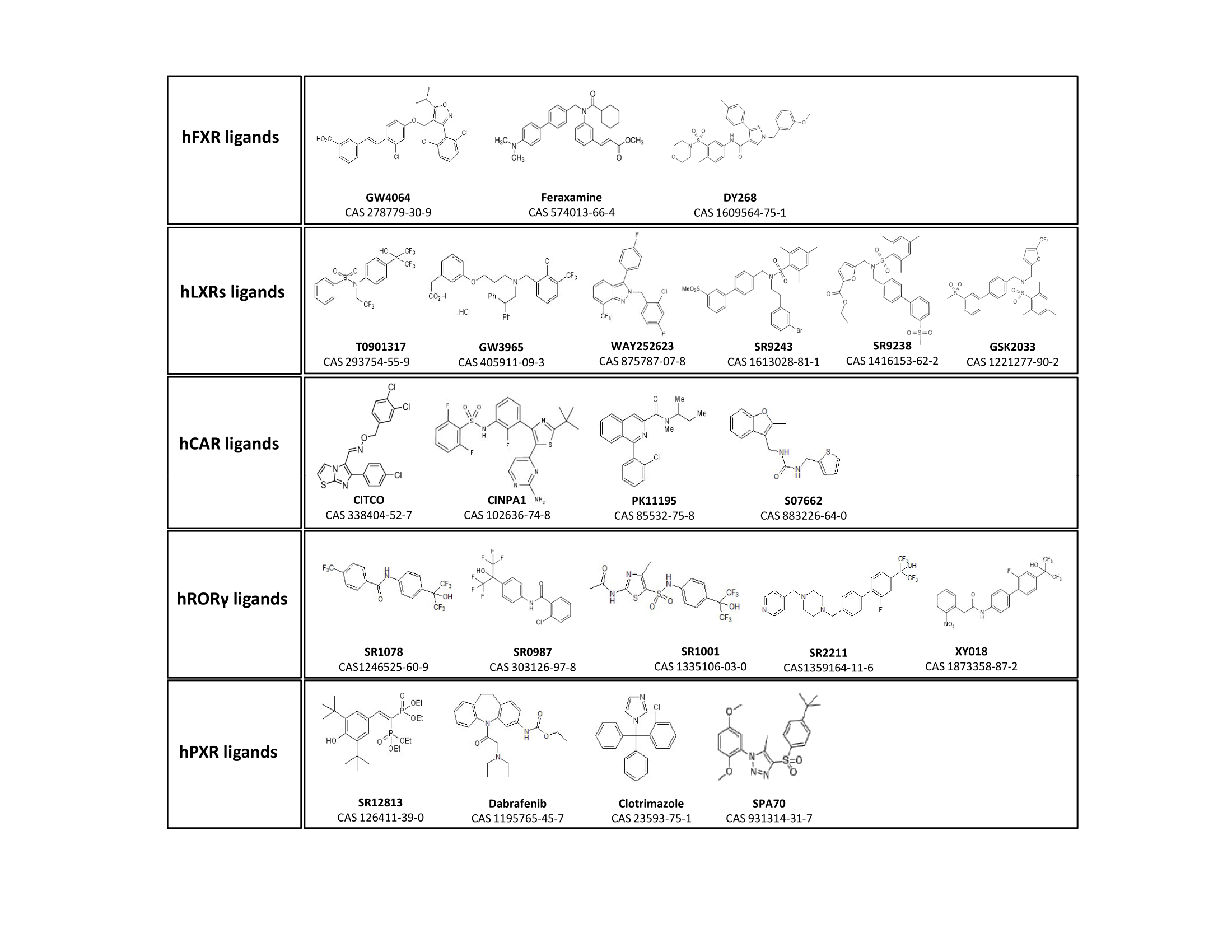

Supplement: Figure S1 — Chemical structure of tested pharmaceutical compounds. [file Image_1.tiff]
